# Gallbladder function predicts subsequent biliary complications in patients with common bile duct stones after endoscopic treatment?

**DOI:** 10.1186/s12876-018-0762-6

**Published:** 2018-02-27

**Authors:** Tzung-Jiun Tsai, Hoi-Hung Chan, Kwok-Hung Lai, Chih-An Shih, Sung-Shuo Kao, Wei-Chih Sun, E-Ming Wang, Wei-Lun Tsai, Kung-Hung Lin, Hsien-Chung Yu, Wen-Chi Chen, Huay-Min Wang, Feng-Woei Tsay, Huey-Shyan Lin, Jin-Shiung Cheng, Ping-I Hsu

**Affiliations:** 1 Division of Gastroenterology and Hepatology, Department of Internal Medicine, Kaohsiung Veterans General Hospital, Kaohsiung, Taiwan, Republic of China; 2 School of Medicine, National Yang-Ming University, Taipei, Taiwan, Republic of China; 3 Department of Health-Business Administration, Fooyin University, Kaohsiung, Taiwan, Republic of China; 4 Department of Biological Sciences, National Sun Yat-sen University, Kaohsiung, Taiwan, Republic of China; 5 Department of Business Management, National Sun Yat-sen University, Kaohsiung, Taiwan, Republic of China; 6 College of Pharmacy and Health Care, Tajen University, Pingtung city, Taiwan, Republic of China

**Keywords:** Endoscopic retrograde cholangiopancreatography, Gallbladder dyskinesia, Gallstones, Recurrent biliary complications

## Abstract

**Background:**

In patients with common bile duct stones (CBDS) and intact gallbladder, further management for the gallbladder after the CBDS clearance is still controversial. The relationship between gallbladder motility and the biliary complications were seldom discussed. Our study is to predict the subsequent biliary complications by gallbladder function test using fatty meal sonography (FMS) in patients with CBDS who had been treated by endoscopic retrograde cholangiopancreatography (ERCP).

**Methods:**

Patients with an intact gallbladder and CBDS after endoscopic clearance of bile duct were enrolled. Patients received a fatty meal sonography after liver function returned to normal. The fasting volume, residual volume, and gallbladder ejection fraction (GBEF) in FMS were measured. Relationships of patients’ characteristics, gallbladder function and recurrent biliary complication were analyzed.

**Results:**

From 2011 to 2014, 118 patients were enrolled; 86 patients had calculus gallbladders, and 32 patients had acalculous gallbladders. After a mean follow- up of 33 months, 23 patients had recurrent biliary complications. Among 86 patients with calculus gallbladder, 15 patients had spontaneous clearance of gallbladder stones; 14 patients received cholecystectomy due to acute cholecystitis or recurrent colic pain with smooth postoperative courses. In the follow up period, six patients died of non-biliary causes. The GBEF is significant reduced in most patients with a calculus gallbladder in spite of stone color. Calculus gallbladder, alcohol drinking and more than one sessions of initial endoscopic treatment were found to be the risk factors of recurrent biliary complication.

**Conclusions:**

Gallbladder motility function was poorer in patients with a calculus gallbladder, but it cannot predict the recurrent biliary complication. Since spontaneous clearance of gallbladder stone may occur, wait and see policy of gallbladder management after endoscopic treatment of CBDS is appropriate, but regular follow- up in those patients with risk factors for recurrence is necessary.

## Background

Endoscopic sphincterotomy (EST), endoscopic papillary balloon dilation (EPBD), and endoscopic papillary large balloon dilation (EPLBD) are commonly used methods to enlarge the biliary orifice and remove common bile duct stones (CBDS) [1, 2]. In patients with CBDS and an intact gallbladder, the management of the gallbladder after endoscopic clearance of the bile duct is controversial. Some studies suggest that elective cholecystectomy after endoscopic clearance of CBDS may reduce the late biliary complications [3–5], but other studies have not confirmed the same benefits [6–8]. Tsujino et al. found that patients either with cholecystectomy before EPBD or with a calculus gallbladder had higher rate of CBDS recurrence than those with elective cholecystectomy after EPBD or an acalculous gallbladder (10.8% and 15.6% vs. 2.4% and 5.9%, respectively) [9]. In our previous study, patients with calculus gallbladder exhibited a higher incidence of an overall delayed biliary complications than those with acalculous gallbladder and cholecystectomy both before and after endoscopic treatment for CBDS. However, the incidence of recurrent CBDS in patients with calculus gallbladder was similar to that in the cholecystectomized patients, but higher than in patients with acalculous gallbladder [10]. Since slow biliary emptying contribute to recurrent CBDS even after endoscopic sphincterotomy [11], the gall bladder left in situ may be able to wash away bile and prevent recurrence or flush out newly produced stones [12]. Although the filling and emptying of the gallbladder may be impaired in patients with gallstones [13], improved gallbladder emptying and reduced lithogenicity of bile have been reported after endoscopic sphincterotomy [14, 15]. Sugiyama et al. found that EPBD did not affect gallbladder motility in the long-term (five years) [16]. However, the relationship between gallbladder status, motility and recurrent biliary complications after endoscopic treatment has seldom been discussed.

The aim of our study is to evaluate the gallbladder function and outcome of patients with CBDS and an intact gallbladder after endoscopic clearance of the bile duct, and to evaluate the relationship between the gallbladder motility, gallstone status, and other factors that affect the recurrence of biliary complications.

## Methods

This study was approved by the Institutional Review Board of Kaohsiung Veterans General Hospital and was performed according to the Helsinski Declaration. The protocol was registered in the Government Research Bulletin according to the law of Taiwan. Informed written consent was obtained from all participants in this study.

### Patients

Patients with an intact gallbladder and CBDS, including patients with biliary pancreatitis, who had received endoscopic clearance of bile duct and refused for elective cholecystectomy, were enrolled. Patients who had recurrent CBDS, association with intrahepatic stones or malignant diseases, first detected recurrent biliary complication during the follow-up period (within six months) after clearance of bile duct, elective cholecystectomy subsequently for non-biliary cause, or refusals of follow-up were excluded.

### Endoscopic treatment

All the endoscopic treatments were performed under local anesthesia of the pharynx with 10% xylocaine, intramuscular injection with 40 mg hyosine-N butybromide and 25-50 mg meperidine were administered as premedication. Endoscopic retrograde cholangiopancreatography (ERCP) was performed in the standard manner using a side-view endoscope (JF240, JF260; Olympus Optical Corporation, Tokyo, Japan). End-view endoscope (GIF1T, GIFXQ, Olympus Optical Corporation, Tokyo, Japan) was used in patient with prior Billroth II gastrectomy. Endoscopic large balloon dilation (CRE balloon 5.5 cm in length,1–1.2 cm/1.2–1.5 cm/1.5–2.0 cm in diameter; Boston Scientific Corp, Ireland) usually performed to enlarge the papillary orifice. Using fluoroscopic and endoscopic guidance, the balloon was inflated with diluted contrast solution up to the optimal size for 2–6 min according to the patients’ condition and tolerance. In order to minimize the risk of perforation, the size of balloon should not be exceeded the diameter of the CBD [17]. In the patients with difficult deep cannulation, precut followed by full sphincterotomy using needle knife sphincterotome(KD-V451 M, Olympus Co. Japan) and conventional sphincterotome (Truetome 39, Boston Scientific Crop, Ireland) were performed. After enlargement of papillary orifice, the CBDS were retrieved by basket (FG V22PR, FGV21PR, Olympus Co. Japan) until completely clearance of bile duct. If the stones were larger than the distal bile duct, they were fragmented by lithotripter (BML-V232QR-30, Olympus Co. Japan) before extraction.

### Stone color stratification

According to natural history of gallstones and CBD stones, we classified the stones into primary and secondary CBD stone according to the stone color [18, 19]. The secondary CBD stone were white cholesterol or black pigment stone, which forms primary in gallbladder and secondary migrate into CBD. The primary stone was brown pigment stone, which usually forms primary in CBD.

### Follow-up studies

Patients were followed in a special clinic every two weeks soon after discharge. Patients received a fatty meal sonography (FMS) to evaluate the gallbladder function when their liver function returned to normal. Patients were then scheduled for follow-up visits every three months. During each visit, a blood sample was taken for liver function test every three months. Abdominal sonogram was performed every six months or at the time of abnormal liver function test or clinical symptoms suggesting recurrent biliary complications. Further image study, such as CT scan, magnetic resonance cholangiopancreatography (MRCP) or ERCP would be performed if recurring biliary symptoms, abnormal liver function test, or abdominal sonography suggested recurrent stones. Cholecystectomy would be advised if biliary symptoms indicated cholecystitis.

### Fatty meal sonography

After fasting for eight hours, routine abdominal sonography was performed to measure the fasting gallbladder volume. The volume of gallbladder was measured by the ellipsoid method as described by Dodds et al. [20]. A fatty meal with two fried eggs and 250 mL of full milk (fat 28 g,protein 22 g, carbohydrate 12 g, total 388 Kcal) was taken by the patients after measurement of fasting gallbladder volume [21]. Repeated gallbladder volume measurement every 15 min until 90 min were done after the intake of fatty meals. The parameters, including the fasting volume, residual volume, maximal gallbladder ejection fraction (EF) and EF at 30 min after the fatty meals were measured for analysis.

### Statistics

The clinical data was analyzed by IBM SPSS v.20. All the values were expressed as mean ± SD or frequency with percentage. Differences between two groups were analyzed, and two-tailed Student t test was used for continuous data and chi-square test for categorical data. Univariate and multivariate Cox regression analyses were used to evaluate the factors affecting the recurrent biliary complications. *P* < 0.05 was considered to be statistically significant.

## Results

**Fig. 1:**
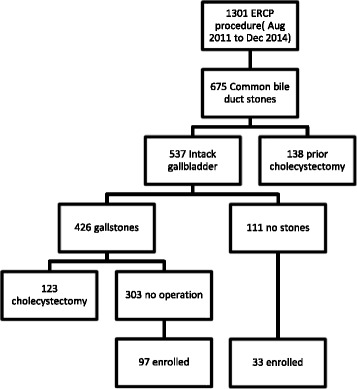
Algorithm of patients enrolled to study

From August 2011 to December 2014, 1301 ERCP procedures were performed at Kaohsiung Veterans General Hospital. A total of 675 patients who were diagnosed as having CBDS based on their clinical symptoms, laboratory tests, and image studies, received endoscopic treatment to clear the bile duct. Of these 675 patients, 138 patients had previously received a cholecystectomy, and 537 had an intact gallbladder. One hundred and thirty patients initially agreed to join this study, but 12 were excluded. These include three patients with intrahepatic duct stones, seven refusals of subsequent follow-up, one with newly developed cancers within six months of follow-up, and one with recurring biliary complication before receiving gallbladder function test (Fig. 1).

Among the 118 patients, 76 were male and 42 patients were female. The mean age was 63.6 ± 17.5 years old. Thirty one patients had habitual smoking, and 18 patients had at least a drink per week. Concomitant diseases included chronic lung disease in seven patients, cerebrovascular disease in seven patients, cardiovascular disease in 53 patients, renal failure in seven patients, a history of cancer disease after remission or curative treatment in 12 patients, chronic liver disease in 12 patients, and diabetes mellitus in 25 patients. 24 patients were acute biliary pancreatitis, but refused cholecystectomy after well explanation. Gallbladder stones were identified in 86 patients by an abdominal sonogram or a CT before endoscopic treatment. Forty-five patients had a juxtapapillary diverticulum. The mean common bile duct diameter was 1.2 ± 0.4 cm. Five patients received endoscopic sphincterotomy (EST), while 113 patients received EPBD to enlarge the papillary orifice. In the patients who received EPBD, the mean diameter of the balloon was 1.1 ± 0.2 cm. Twelve patients received mechanical lithotripsy to retrieve stones. Twenty-three patients did not have gross stone retrieval after enlargement of the papillary orifice even though CBDS had been previously reviewed by other images. 51 of 95 extracted stones were brown pigment in type. The mean duration of the procedure was 49.2 ± 22 min. The procedures were successful in the first session for 110 patients. Four patients developed complications, including mild acute pancreatitis in three patients and fever with bacteremia after endoscopic treatment in one, and all patients recovered after conservative treatment for those complications.

Patients were followed for a median of 33 months (15–56 months). Recurrent biliary complications occurred in 23 patients (22 had GB stones and the other one without). Five patients suspected recurrent CBDS based on the symptoms and image findings at intervals of 12 to 31 months from the initial procedure. Two patients were confirmed to have stones and retrieved endoscopically; no gross stone was found during the endoscopic procedure in the other three patients. Two patients (one with gallbladder stones and the other without) developed acute cholangitis at eight and 28 months following the initial procedure and their symptoms subsided after conservative treatment. Ten patients developed acute cholecystitis at three to 28 months after initial endoscopic treatment. Although one of the ten patients had a ruptured gallbladder, he recovered smoothly after cholecystectomy. Six patients suffered from recurrent biliary pain at two to 22 months following initial endoscopic treatment, and four of them received elective cholecystectomy; all were well after their operation. Two patients with recurrent biliary pain refused elective cholecystectomy and were well after conservative treatment (Table 1).

**Table 1:** Patients’ characteristics

Parameter	Number (%)
Gender (male)	76 (64.4%)
Age (mean±SD)	63.6±17.5 years
Smoking	31 (26.3%)
Alcohol drinking	18 (15.3%)
Concomitant disease	
Chronic lung diseases	7 (5.9%)
Cerebrovascular diseases	7 (5.9%)
Cardiovascular diseases	53 (44.9%)
Renal failure	7 (5.9%)
Cancer diseases	12 (10.2%)
Liver disease	12 (10.2%)
Diabetes mellitus	25 (21.2%)
Juxtapapillary diverticulum	45 (38.1%)
Gallbladder stones	86 (72.9%)
Biliary pancreatitis	24 (20.3%)
CBD diameter (mean±SD)	1.2±0.4 cm
Number of CBD stones (no/single /multiple)	23/59/36 (20/50/30)
Largest CBD stones size (mean±SD)^a^	0.9±0.4 cm
Endoscopic procedures (EST/EPBD)	5/113 (4/96)
Mean balloon diameter for EPBD (mean±SD)	1.1±0.2 cm
Mechanical lithotripsy	12 (10.2%)
Sessions for bile duct clearance(> 1 session)	8 (6.8%)
Stone color^a^	
Cholesterol and black pigment stones	44 (46%)
Brown pigment stones	51 (54%)
Mean ERCP procedure time (mean±SD)	49.2±22.2 min
Postprocedural complications	4 (3.4%)
Follow up time (median, range) months	33 (15 to 56)
Recurrent biliary complications	23 (19.5%)
Spontaneous passage of gallstones	15 (15/86 = 17.4%)
Death during follow up	6 (5.1%)

CBD: common bile duct, EST: endoscopic sphincterotomy, EPBD: endoscopic papillary balloon dilation

^a^23 patients with stone pass-out were not included

Spontaneous clearance of stone from the gallbladder of 15 patients was confirmed by a subsequent abdominal sonogram at a median of 12 months (range one to 31 months) after endoscopic treatment. No common bile duct stones were detected in these 15 patients. Eight patients had a single gallbladder stone, and seven patients had multiple gallbladder stones. The mean size of the stones was 0.8±0.4 cm (range 0.2 to 1.8 cm). Fifteen patients were symptom-free during the follow-up period. No new stone was found by sonography during the follow-up period in patients without stone in their gallbladder. FMS was successfully conducted in 110 patients, and failed in eight patients owing to either small contracted gallbladders filled with stones or gas blockages. One patient died of rectal cancer at 14 months following the endoscopic treatment; the other one patient was found to have lung cancer at seven months later and died at 22 months. Four patients died from renal failure, pneumonia, chronic hepatic failure, and heart failure, respectively.

Comparing with gallbladder stone, patients without it had a significantly higher incidence of chronic lung disease, a larger common bile duct diameter, larger stones, a higher incidence of the non-visualization of the gallbladder in ERCP, a larger mean diameter of the dilating balloon used, a higher frequency to use mechanical lithotripsy, and a larger percentage of gallbladder contraction of > 50% at 30 min after a fatty meal (Table 2).

**Table 2:** Differences between the patients with and without gallstones

	Gallbladder stones (+)	Gallbladder stones (−)	*P*
Gender (male/female)	55/31	21/11	0.866
Age (mean± SD, years)	62.5±17.9	66.5±16.6	0.276
Smoking (yes/no)	19/67	12/20	0.091
Alcohol drinking (yes/no)	13/73	5/27	0.946
Concomitant diseases			
Chronic lung disease (yes/no)	2/84	5/27	0.016
Cerebrovascular diseases (yes/no)	4/82	3/29	0.387
Cardiovascular diseases (yes/no)	37/49	16/16	0.016
Renal failure (yes/no)	7/79	0/32	0.187
Cancer diseases (yes/no)	10/76	2/30	0.509
Liver disease (yes/no)	7/79	5/27	0.232
Diabetes mellitus (yes/no)	15/71	10/22	0.103
JPD (yes/no)	29/57	16/16	0.106
CBD diameter (mean±SD, cm)	1.2±0.4	1.4±0.4	0.017
CBDS pass-out (yes/no)	18/68	5/27	0.518
Brown pigment stones (yes/no)	38/30	13/14	0.495
Largest CBDS size (mean±SD, cm)	0.8±0.4	1.0±0.5	0.016
CBDS number (single/multiple)^a^	43/25	16/11	0.719
Mean balloon diameter for EPBD (mean±SD)^b^	1.0±0.2	1.2±0.3	0.002
Mechanical lithotripsy (yes/no)	4/82	8/24	0.003
Sessions for bile duct clearance (1/> 1)	80/6	30/2	1.000
Non-visualization of gallbladder in ERCP (yes/no)	24/62	19/13	0.002
Fatty meal sonography^a^			
Fasting volume(ml)	20.4±18.8	23.0±21.9	0.557
Residual volume (ml)	5.2±5.3	6.0±11.8	0.624
Ejection fraction (%)	48.9±36.0	63.2±33.1	0.070
Ejection fraction at 30 min (≥50% vs < 50%)	39/44	20/7	0.014

*JPD*: juxtapapillary diverticulum, *CBD*: common bile duct, *CBDS*: common bile duct stone, *EPBD*: endoscopic papillary balloon dilation

^a^8 patients with small contracted gallbladder or gas block were excluded

^b^Only the 113 patients who received EPBD were included

According to the univariate Cox regression analysis, the presence of gallbladder stones, the drinking of alcohol, renal failure, and more than one sessions of initial treatment for bile duct clearance significantly affect recurrent biliary complications (*P* < 0.05, Table 3). Moreover, the multivariate Cox regression analysis revealed that only the presence of gallbladder stones, the drinking of alcohol and more than one sessions of initial treatment for bile duct clearance are significantly associated with recurrent biliary complications (*P* < 0.05, Table 4). No significant differences existed between any of the parameters of gallbladder function by FMS between patients with different stone colors, patients with recurrent biliary complications, and patients who spontaneously passed their gallbladder stones.

**Table 3:** Factors affecting the recurrent biliary complications (univariate analysis)

Factors	Risk ratio (95% CI)	*P*
Gender (male/female)	1.53 (0.60–3.89)	0.369
Mean age	1.00 (0.99–1.03)	0.860
Smoking (yes/no)	2.19(0.96–5.01)	0.062
Alcohol drinking (yes/no)	2.49 (1.02–6.05)	0.045
Concomitant diseases		
Chronic lung disease (yes/no)	1.70(0.40–7.27)	0.473
Cerebrovascular diseases (yes/no)	1.68(0.39–7.18)	0.482
Renal failure (yes/no)	3.19(1.09–9.39)	0.035
Cancer diseases (yes/no)	1.52(0.45–5.11)	0.501
Liver disease (yes/no)	2.35(0.79–6.98)	0.123
Diabetes mellitus (yes/no)	1.03(0.38–2.27)	0.958
JPD (yes/no)	1.05(0.45–2.42)	0.888
Gallbladder stones(Yes/No)	8.83(1.19–65.49)	0.033
Mean CBD diameter	2.26(0.83–6.180	0.113
Mean balloon diameter for EPBD^a^	3.10(0.50–1900)	0.222
Mechanical lithotripsy (yes/no)	1.06(0.25–4.58)	0.935
Brown pigment stones (yes/no)	1.16(0.47–2.84)	0.748
Sessions for bile duct clearance(1/> 1)	0.32(0.11–0.93)	0.037
Non-visualization of gallbladder in ERCP(yes/no)	1.96(0.73–5.29)	0.184
Fatty meal sonography^b^		
Fasting volume	1.01(0.99–1.03)	0.596
Residual volume	1.02(0.98–1.06)	0.324
Ejection fraction	1.00(0.99–1.01)	0.986
Ejection fraction at 30 min (≥50% vs < 50%)	0.70(0.31–1.59)	0.400

*JPD*: juxtapapillary diverticulum, *CBD*: common bile duct, *EPBD*: endoscopic papillary balloon dilation. *ERCP*: endoscopic retrograde cholangiopancreatography

^a^Only the 113 patients who received EPBD were included

^b^8 patients with small contracted gallbladder or gas block were excluded

**Table 4:** Multivariate analysis of the factors affecting recurrent biliary complications

Factors	Risk ratio (95% CI)	*P*
Gallstones (yes/no)	8.42(1.12–63.14)	0.038
Alcohol drinking (yes/no)	2.95(1.14–7.68)	0.026
Renal failure (yes/no)	1.50(0.49–4.67)	0.480
Sessions for bile duct clearance (1/> 1)	0.26(0.08–0.84)	0.024

## Discussions

Reduced gallbladder motility is widely recognized as an important factor in the formation of cholesterol stones [22], but the role of the gallbladder function in the formation of pigment stones is controversial [23–25]. Brown stones form secondary to stasis and anaerobic bacterial infection in any part of the biliary tree including the gallbladder [26, 27]. A higher percentage of our patients with calculus gallbladder than that of the acalculous gallbladder, had suboptimal gallbladder motility (53.3% vs. 25.9%), so gallbladder motility should be considered as a factor in the formation of gallbladder stone.

Following the endoscopic treatment of CBDS, recurrent biliary complication occurred in 3–21% of patients after EST [12] and in 5–25% of patients after EPBD [28]. Calculus gallbladder was identified as one of the factors that is responsible for such complications [29]. EPLBD, using balloon ≥12 mm, is a safe and effective method in facilitating the removal of CBDS as seen, but is not a sphincter-preserving procedure [30]. Sphincter-preserving methods such as EPBD, using the conventional 8 mm balloon should be suitable for patients with secondary CBDS that migrates from the gallbladder. EPLBD and a full EST can facilitate the biliary drainage of the bile duct and are suitable procedures for patients with primary CBDS or a prior cholecystectomy [30, 31]. Although gallbladder motility improved only temporarily after endoscopic papillary dilation using a conventional 8 mm balloon [16, 32], improvement of gallbladder emptying and facilitation of the spontaneous passage of gallbladder stones after EST have been reported [14, 33]. In this study, even gallbladder ejection in patients with a calculus gallbladder was inferior to that in patients with an acalculous gallbladder, and 46.7% of the former maintained optimal gallbladder ejection. The presence of gallbladder stones rather than the gallbladder EF was the factor affected the late biliary complications in this study.

Non-filling of the gallbladder may be an indication for cholecystectomy [34, 35]. Non-filling of the gallbladder may lead to failure of gallbladder contraction. However, bile stasis with subsequent sepsis and carcinoma is questionable, and no strong evidence supports the beneficial effect of cholecystectomy in patients with non-filling gallbladder [7, 36, 37]. Although 8.4% (10/118) of patients developed acute cholecystitis (including one with gallbladder rupture) after endoscopic treatment for CBDS, all patients recovered completely after their operation. In addition, fifteen patients (12.7%) were found to have been spontaneously passed their gallbladder stones in the follow-up period. Endoscopic treatment of the biliary sphincter by either EST or endoscopic sphincterotomy plus large balloon dilation (ESLBD) may increase the gallbladder motility, facilitating the spontaneous passing of gallstones and increasing the risk of recurrent biliary complications, particularly gallbladder complications [38]. Except in cases of gallbladder-related complications, elective cholecystectomy does not help to prevent recurrent CBDS or cholangitis [29, 39]. Other studies have shown that prior cholecystectomy may be a factor in causing the recurrent CBDS [12, 40, 41]. These contradictory results raise a question regarding who will benefit from elective cholecystectomy following endoscopic treatment for CBDS. Some studies claimed that prophylactic cholecystectomy is not required in patients with acalculous gallbladder following endoscopic clearance of the bile duct [29], but elective cholecystectomy in patients with calculus gallbladder following endoscopic treatment is recommended owing to the risk of subsequent recurrent biliary complications [42, 43]. However, most relevant studies that strongly recommend routine cholecystectomy neglect the evidence of the spontaneous clearance of the gallbladder following endoscopic treatment. Although a calculus gallbladder is identified as a significant risk factor for recurrent biliary complications in this study, the rate of acute cholecystitis as a late complication was 8.4% (10/118), whereas the rate of asymptomatic spontaneous clearance of gallbladder stones was 12.7% (15/118). All patients in the current study were regularly followed at our clinic and were alert for recurrent symptoms. As a result, the possible recurrent complications can be detected early and managed properly [44]. Therefore, the wait-and-see policy for the patients with simple gallbladder stones may also be applicable for the patients with concurrent gallbladder stones and CBDS following endoscopic treatment. Surgical intervention should be conducted only on patients with recurrent gallbladder- related complications to prevent an unnecessary cholecystectomy, particularly for the aged patients and patients with a high likelihood of the spontaneous emptying of gallbladder stones, including those with small stones, a wide cystic duct, low or a small angle of cystic duct insertion with CBD [45–47].

FMS is a simple and accurate noninvasive test to determine gallbladder size and contraction [20, 48]. A simple fatty meal can induce a neurocephalic and hormonal response, and it is more physiological than using cholecystokinin injection in testing the gallbladder emptying [49]. A large fasting or residual gallbladder volume, and a low ejection fraction at 30 or 45 min have been reported to be present in patients with calculus gallbladder [50–53]. The parameters of gallbladder motility may be influenced by body weight, age, sex, the patency of the cystic duct and the biliary tract, and the method of testing, so cutoff values have ranged from 35% to 80% in the literature [54, 55]. 50% is used as the cutoff value of GBEF at 30 min because our patients had previously received endoscopic treatment of the sphincter of Oddi. Although the incidence of abnormal GBEF at 30 min is significantly higher in calculus gallbladders than acalculous gallbladders, no variation existed in this respect among the patients with late biliary symptoms, patients with spontaneously passed gallbladder stones and patients who fell in neither group. Fifty four percent of extracted stones were brown pigment stones which were produced primarily in the bile duct. This fact also may explain failure to identify the relationship between gallbladder function and recurrent biliary complications in our patients. According to simultaneous fatty meal scintigraphy and sonography, the postprandial gallbladder can handle up to six times its basal volumes within a period of 90 min in healthy subjects, but the turnover of bile is markedly reduced in patients with a calculus gallbladder [56]. Since the gallbladder function is complex and cannot be reliably measured by FMS or scintigraphy alone to predict the symptoms or outcome of a cholecystectomy [57, 58], whether simultaneous scintigraphy and sonography is a better method than either requires further investigation to determine.

Along with a calculus gallbladder, the drinking of alcohol at least weekly and initial clearance of the bile duct with more than one session of endoscopic treatment affect recurrent biliary complications in this study. The effect of alcohol on the gallbladder and the biliary tract are controversial. Peoples who frequently consume alcohol are less likely to suffer from gallstones disease [59]. Alcohol has been found to inhibit activity of the sphincter of Oddi [60], alcohol may have affected the residual function of the sphincter of Oddi or emptying of the bile duct in our patients even following endoscopic treatment, resulting in delayed biliary emptying and biliary complications [11]. Incomplete clearance of the bile duct in one session significantly affects recurrent biliary complications, and most patients who required a second-look procedure, usually had multiple or difficult stones. Some radiological undetected small stone fragments may have been left in the bile duct, and their migration may cause recurrent symptoms.

This study has some limitations. The follow-up time was not long enough to observe the rate of re-recurrent biliary complications in patients who had undergone a cholecystectomy. Most of the stones in our patients were mixed black and brown, so differentiating between secondary and primary bile duct stones was difficult. Our patients were acutely ill before endoscopic treatment, so no FMS was performed before endoscopic treatment for baseline reference. Owing to blockage by gas or stone, FMS was not successfully performed in all patients. A further long-term study is required to evaluate the clinical significance of gallbladder function and the effect of cholecystectomy in these patients.

## Conclusions

Gallbladder ejection fraction was poorer in patients with gallbladder stones, but it could not predict further recurrent biliary complications. Alcohol restriction would probably help to reduce recurrent biliary symptoms. Since spontaneous clearance of gallbladder stone may occur, wait and see policy of gallbladder management after endoscopic treatment of CBDS is appropriate, but regular follow- up in those patients with risk factors for recurrence is necessary. Cholecystectomy is warranted only in patients with recurrent gallbladder-related symptoms.

## Abbreviation

CBD: common bile duct
CBDS: common bile duct stone
EF: ejection fraction
EPBD: endoscopic papillary balloon dilation
EPLBD: endoscopic papillary large balloon dilation
ERCP: endoscopic retrograde cholangiopancreatography
ESLBD: endoscopic sphincterotomy plus large balloon dilation
EST: Endoscopic sphincterotomy
FMS: fatty meal sonography
GBEF: gallbladder ejection fraction
MRCP: magnetic resonance cholangiopancreatography
